# Integrative preclinical strategy for pharmacokinetic profiling and candidate selection of 3-hydroxypropanamidines: a promising antimalarial class

**DOI:** 10.1186/s12936-026-05837-9

**Published:** 2026-03-10

**Authors:** Alena Moritz, Bastian Hirn-Derksen, Saskia Klein, Jana Held, Sergio Wittlin, Matthias Rottmann, Thomas Kurz, Bjoern B. Burckhardt

**Affiliations:** 1 Individualized Pharmacotherapy, Institute of Pharmaceutical and Medicinal Chemistry, University of Münster, Corrensstr. 48, 48149 Münster, Germany; 2 University Hospital Münster, Albert Schweitzer Campus 1, 48149 Münster, Germany; 3 Institute of Pharmaceutical and Medicinal Chemistry, Heinrich Heine University Düsseldorf, Universitätsstr. 1, 40225 Düsseldorf, Germany; 4 Institute of Tropical Medicine, Eberhard Karls University Tübingen, Wilhelmstr. 27, 72074 Tübingen, Germany; 5 German Center for Infection Research, Partner Site Tübingen, 72074 Tübingen, Germany; 6 Centre de Recherches Medicales de Lambaréné, B.P. 242, Lambaréné, Gabon; 7 Swiss Tropical and Public Health Institute, Kreuzstr. 2, 4123 Allschwil, Switzerland; 8 Faculty of Science, University of Basel, 4003 Basel, Switzerland

**Keywords:** Malaria, Drug development, Pharmacokinetics, Preclinical, Physiologically based pharmacokinetic modeling

## Abstract

**Background:**

3-Hydroxypropanamidines represent a promising novel, highly lipophilic class of oral antimalarial drugs developed in response to the urgent need for new antimalarials due to the increasing resistance of Plasmodia. A preclinically guided selection approach was conducted, combining optimized in silico, in vitro/ex vivo, and in vivo assays to guide pharmacokinetic-driven compound selection.

**Methods:**

Preliminary sorting was enabled by several in vitro/ex vivo assays (intestinal permeability, plasma protein binding, blood-to-plasma ratio, and microsomal stability), adapted for high lipophilicity. To challenge this sorting, the most promising and the least promising 3-HPAs were selected for further in vivo studies in *Plasmodium berghei*-infected mice (concentration–time profiles and racemate separation). Finally, a physiologically based pharmacokinetic model was built for the overall most promising 3-HPA to gain initial insights into its pharmacokinetic behavior in humans.

**Results:**

The most hydrophilic compounds, TKK130 (a low-extraction drug) and SAKK381 (a high-extraction drug), presented the most promising in vitro/ex vivo pharmacokinetic profiles (i.e., the highest intestinal permeability and unbound plasma fraction). In particular, TKK130 was favorable because the blood-to-plasma ratio indicated a slight preference for distribution into red blood cells. One of the most lipophilic 3-HPAs, SAKK394 (a low-extraction drug), exhibited the poorest in vitro/ex vivo profile. In vivo, TKK130 demonstrated a sustained pharmacokinetic profile with the highest dose-adjusted total exposure over time, the lowest enantioselective clearance, and a 100% cure rate without signs of toxicity. The physiologically based pharmacokinetic model for the most promising TKK130 demonstrated a good fit to the in vivo data. Extrapolation to humans enabled the first human pharmacokinetic prediction, which was compared to the profile of lumefantrine. Profiles in adults were characterized by high interindividual variability (e.g., total exposure of 814–3856 ng/mL h) and food effects (e.g., total exposure (fasted 1846 ng/mL h vs. fed 3407 ng/mL h)).

**Conclusions:**

TKK130 was identified as the most favorable compound of the novel antimalarial 3-hydroxypropanamidines because of its encouraging pharmacokinetic profile, combined with its excellent in vivo efficacy and lack of observed toxicity in mice. TKK130 is a promising candidate for further preclinical and clinical development.

**Graphical Abstract:**

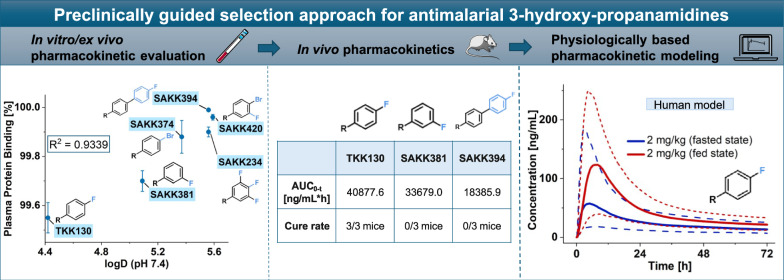

**Supplementary Information:**

The online version contains supplementary material available at 10.1186/s12936-026-05837-9.

## Background

High resistance rates of Plasmodia to known antimalarials still cause a major burden in effective malaria therapy, as reflected by 597,000 deaths worldwide attributed to malaria in 2023 [1]. With the introduction of vaccines, a decisive step has been taken toward better prevention of the disease. However, until these are fully effective and long-term achievements can be assessed, chemotherapy will continue to play a central role in malaria treatment [2–6].

Nevertheless, developing new potent antimalarials is challenging, as demonstrated by the fact that only one compound (tafenoquine, 2018) has been approved by the U.S. Food and Drug Administration (FDA) during recent decades [7, 8]. According to the nonprofit organization Medicines for Malaria Venture (MMV), 14 research projects are currently developing new antimalarial lead structures, while six promising compounds are in the preclinical phase [9]. Research teams are encountering profound challenges in the preclinical development phase, including the low solubility of drug candidates (i.e., GSK701 and MMV609) and limitations in the prediction of human pharmacokinetics (PK) (GSK484) [9, 10].

The implementation of in vitro, ex vivo, and in vivo assays for PK evaluation in preclinical studies has resulted in a notable reduction in drug failure in clinical trials due to poor physicochemical or pharmacokinetic characteristics, ranging from 30–40% in 1990 to 10–15% in 2022 [11, 12]. In recent years, in vitro and ex vivo assays have undergone considerable advancements, yielding more meaningful datasets and improved scalability. These improvements are reflected by the U.S. FDA in 2022 by issuing the Modernization Act 2.0, which now permits preclinical drug evaluations to be conducted without the need for mandatory animal testing when certain alternatives, i.e., in vitro/ex vivo assays and computer models, are used [13–15]. Despite improvements in these assays, suboptimal pharmacokinetics remain a reason for the failure of potential drug candidates, and further assay optimization is needed to overcome this limitation [12, 16]. For the PK evaluation of highly lipophilic antimalarials, guidelines for appropriate and physiologically relevant analyses are currently lacking. Consequently, highly lipophilic compounds are at risk of failure during the drug development process.

A promising novel class of oral antimalarial compounds are the 3-hydroxypropanamidines (3-HPAs) [17, 18]. Owing to the high lipophilicity of 3-HPAs (logD values >4), a comprehensive preclinical strategy was implemented, integrating adapted in vitro/ex vivo assays, in vivo studies, and physiologically based pharmacokinetic (PBPK) modeling. This concept facilitated pharmacokinetic-driven preselection of the most promising new compounds and enabled the investigation of in vitro/ex vivo to in vivo transferability.

## Methods

### In vitro/ex vivo pharmacokinetic evaluation

#### Intestinal permeability

Passive diffusion was determined via Kerski diffusion cells with PermeaPad^®^ membranes (PHABIOC GmbH, Karlsruhe, Germany) [19, 20]. The donor chamber was filled with Fasted State Simulating Intestinal Fluid—Version 2 (FaSSIF-V2) buffer spiked with 50 or 100 µg/mL 3-HPA to mimic physiological intestinal conditions [21]. 4% bovine serum albumin (BSA) in phosphate-buffered saline (PBS) at a physiological pH of 7.4 (w/v) was used as the acceptor medium.

The Kerski cells were incubated at 37 °C under constant stirring. Samples were collected from the acceptor chambers at 0, 1, 2, 3, and 4 h in triplicate, and the chambers were replenished with acceptor medium each time. Each sample was precipitated with ice-cold acetonitrile containing an internal standard. The supernatant was evaporated under a gentle stream of nitrogen at 37 °C and reconstituted in HPLC-grade water/acetonitrile (60/40, *v*/*v*). The samples were quantified by high-performance liquid chromatography coupled with tandem mass spectrometry (LC–MS/MS) against a fresh calibration curve of the respective compound in 4% BSA in PBS at pH 7.4 (w/v) (spiked with donor solution). The apparent permeability coefficients (P_app_) were calculated by Eqs. 1–3. The correlation between the measurements and the logD value was determined using Microsoft Excel^®^ Version 2406.

The entire setting was verified earlier using the reference compounds propranolol hydrochloride, verapamil hydrochloride, and metoprolol tartrate by comparing the P_app_ values obtained with their corresponding literature values [22–24].

1 $$Q_{t} = \frac{{C_{t} \times V_{A} + \left( {\mathop \sum \nolimits_{t = 1}^{t} C_{t - 1} } \right) \times V_{R} }}{A}$$

*Q*_*t*_ = Cumulative amount of permeated drug per area at time t (µg/cm^2^), *C*_*t*_ = drug concentration at time point *n* (µg/mL), *C*_*t*−1_ = drug concentration at time point *n*−1 (µg/mL), *V*_*A*_ = volume of acceptor chamber (mL), *V*_*R*_ = removed volume (mL), and *A* = area for permeation (cm^2^).

2 $$J_{ss} = \frac{{\Delta Q_{t} }}{\Delta t \times A}$$

*J*_*ss*_ = Steady-state flux (µg/cm^2^/h), $\Delta Q_{t}$ = difference of *Q*_*t*_ between time points (µg/cm^2^), $\Delta t$ = difference of time points (h), and *A* = area for permeation (cm^2^).

3 $$P_{app} = \frac{{ \frac{{J_{ss} }}{{C_{D} }}}}{3600}$$

*P*_*app*_ = Apparent permeability coefficient (cm/s), *J*_*ss*_ = steady-state flux (µg/cm^2^/h), and *C*_*D*_ = initial donor concentration (µg/mL).

#### Plasma protein binding

Plasma protein binding (PPB) was performed via equilibrium dialysis and the dilution method [25, 26]. A regenerated cellulose membrane was clamped in a 96-well Teflon plate, thereby creating two chambers in each well. The donor chamber was filled with plasma diluted in isotonic saline at ratios of 1:10 and 1:20, and spiked with 25 ng/mL TKK130 or 100 ng/mL (SAKKs). Chosen concentrations were all below the predicted maximum solubility of each compound in water. The acceptor chamber contained the same volume of pure isotonic saline. After 24 h of incubation at a physiological temperature of 37 °C, samples were collected from both chambers.

The donor samples were precipitated with ice-cold acetonitrile (containing an internal standard) at a 1:4 ratio, immediately vortexed, shaken (1000 rpm, 30 min), and centrifuged, and the supernatant was evaporated to dryness at 37 °C under a gentle nitrogen stream before reconstitution. Moreover, the acceptor chamber was mixed with methanol (containing internal standard), resulting in a 0.9% saline/methanol 60/40 (*v*/*v*) solution. The samples were quantified using calibration curves in the respective media. As a quality control measure, the plasma protein binding of itraconazole was determined in each analytical run. The plasma protein binding of the 3-HPAs and itraconazole was assessed in triplicate using LC–MS/MS and calculated according to Eqs. 4–6. The values for the coefficient of determination (*R*^2^) were determined to establish correlations between the results and the logD values using Microsoft Excel^®^.

4 $$f_{u,d(p)} = \frac{{C_{A} }}{{C_{D} }}$$

$f_{u,d(p)}$ = Free fraction in dilution method (plasma), *C*_*A*_ = acceptor concentration (ng/mL), and *C*_*D*_ = donor concentration (ng/mL).

5 $$f_{u,p} = \frac{\frac{1}{D}}{{\left[ {\left( {\frac{1}{{f_{u,d(p)} }}} \right) - 1} \right] + \frac{1}{D}}}$$

$f_{u,p}$ = Fraction unbound in plasma, *D* = dilution level, and $f_{u,d(p)}$ = free fraction from dilution method.

6 $$f_{b,p} = (1 - f_{u,p} ) \times 100$$

$f_{b,p}$= Fraction bound in plasma (%) and $f_{u,p}$ = fraction unbound in plasma.

#### Blood-to-plasma ratio

The blood-to-plasma ratio (K(B/P)) was obtained to determine the distribution of the compounds between the blood and the plasma fraction. Fresh whole blood was spiked with compounds at different concentrations (TKK130 at 1, 5, and 25 ng/mL and SAKKs at 2.5 and 50 ng/mL). Following a 30-min incubation period at 37 °C, the whole blood was divided into the plasma and red blood cell (RBC) fractions by centrifugation at 2000 rpm for 10 min. To obtain references, additive plasma and RBC fractions were obtained from fresh whole blood and spiked with the respective compound at the same concentrations as the samples. The RBC fractions of the samples and references were vortexed and shaken for 10 min to achieve lysis of the red blood cells. The plasma or RBC samples were precipitated with ice-cold acetonitrile (containing internal standard) at a 1:4 ratio, immediately vortexed, shaken (1000 rpm, 30 min), centrifuged, and the supernatant evaporated to dryness at 37 °C under a gentle nitrogen stream before reconstitution. The hematocrit was determined volumetrically. As a reference, the K(B/P) of carvedilol at 25 ng/mL was determined using carvedilol-d5 as the internal standard. K(B/P) was calculated by Eq. 7.

7 $${\mathrm{K}}({\mathrm{B}}/{\mathrm{P}}) = \frac{{\frac{{{\mathrm{area}}\,{\mathrm{ratio}}\,{\mathrm{RBC}}\,{\mathrm{fraction}}}}{{{\mathrm{area}}\,{\mathrm{ratio}}\,{\mathrm{RBC}}\,{\mathrm{reference}}}}}}{{\frac{{{\mathrm{area}}\,{\mathrm{ratio}}\,{\mathrm{plasma}}\,{\mathrm{fraction}}}}{{{\mathrm{area}}\,{\mathrm{ratio}}\,{\mathrm{plasma}}\,{\mathrm{reference}}}}}} \times {\mathrm{H}} + (1 - {\mathrm{H}})$$

K(B/P) = Blood-to-plasma ratio, H = hematocrit, and RBC = red blood cells.

#### Microsomal stability

Microsomal stability was determined in human liver microsomes (HLMs) pooled from 150 donors (Corning, New York, USA). Following the recommendation of the European Medicines Agency (EMA) [27], the cosolvent method was considered with final concentrations of 0.8% acetonitrile and 0.2% dimethyl sulfoxide (Sigma Aldrich, Steinheim, Germany), making it a balanced approach between the physiological conditions of the assay and the required solubility of the highly lipophilic 3-HPAs [28]. During incubation at a physiological temperature of 37 °C while shaking at 450 rpm, the reaction was quenched by the addition of by ice-cold acetonitrile containing an internal standard at 0, 15, 30, 45, and 60 min. The assay was conducted in triplicate. Each analytical run by LC–MS/MS was accompanied by a negative control, a blank control, and propranolol hydrochloride as a reference. The elimination half-life (t_1/2_) and the intrinsic clearance (Cl_int_) were calculated by Eqs. 8 and 9.

8 $$T_{1/2 = } \frac{{{\mathrm{ln}}\left( 2 \right)}}{{k_{e} }}$$

*T*_1/2_ = Elimination half-life (min) and *k*_*e*_ = elimination rate constant (negative slope of the plotted ln compound peak area versus time in min (min^−1^)).

9 $$Cl_{int} = \frac{{{\mathrm{ln}}(2)}}{{T_{1/2 } }} \times \frac{{v_{i} }}{{w_{i} }}$$

*Cl*_*int*_ = Intrinsic clearance (µL/min/mg), *T*_1/2_ = half-life (min), *v*_*i*_ = volume of incubation (µL), and *w*_*i*_ = protein amount in the incubation (mg).

#### Microsomal binding

The nonspecific binding to microsomes was assessed through equilibrium dialysis. The donor medium consisted of 0.5 mg/mL HLMs in potassium phosphate buffer, spiked with 1 µM compound, and an isotonic saline solution was used as the acceptor medium. Samples were obtained from both chambers after 24 h. Donor samples were diluted with 4% BSA in 0.1 M potassium phosphate buffer (w/v) and purified by adding ice-cold acetonitrile containing the internal standard. After shaking and centrifugation, the supernatant was evaporated to dryness under a gentle nitrogen stream at 37 °C, and the residue was reconstituted. Methanol containing the internal standard was added to the acceptor side. Quantification by LC–MS/MS was conducted using calibration curves prepared in their respective media. The microsomal binding was analyzed in triplicate and calculated by Eqs. 10 and 11. The unbound intrinsic clearance (Cl_int,u_) was calculated by Eq. 12 [29]. The hepatic clearance was calculated by Eq. 13, and the hepatic extraction ratio was calculated by Eq. 14.

10 $$f_{b,mic} = \frac{{(D_{Te} - D_{F} ) \times \frac{{V_{e} }}{{V_{i} }}}}{{\left[ {(D_{Te} - D_{F} ) \times \frac{{V_{e} }}{{V_{i} }}} \right] + D_{F} }}$$

$f_{b,mic}$ = Fraction bound in HLMs, $D_{Te}$ = total donor concentration at equilibrium (ng/mL), $D_{F}$ = free concentration of the acceptor (ng/mL), *V*_*e*_ = equilibrium donor volume (µL), and *V*_*i*_ = initial donor volume (µL).

11 $${\mathrm{f}}_{{{\mathrm{u}},{\mathrm{mic}}}} = \left( {1 - {\mathrm{f}}_{{{\mathrm{b}},{\mathrm{mic}}}} } \right)$$

${\mathrm{f}}_{{{\mathrm{u}},{\mathrm{mic}}}} { }$ = Fraction unbound in HLMs.

12 $$Cl_{int,u} = \frac{{Cl_{int} }}{{f_{u,mic} }}$$

*Cl*_*int*,*u*_ = Unbound intrinsic clearance (µL/min/mg), *Cl*_*int*_ = intrinsic clearance (µL/min/mg), and $f_{u,mic}$ = fraction unbound in HLMs.

13 $$Cl_{H} = Q_{H} \times \frac{{\frac{{f_{u,p} }}{K(B/P)} \times Cl_{int,u} }}{{Q_{H} + \frac{{f_{u,p} }}{K(B/P)} \times Cl_{int,u} }}$$

*Cl*_*H*_ = Hepatic clearance (L/min), *Q*_*H*_ = hepatic blood flow (set to 1500 mL/min), *f*_*u*,*p*_ = fraction unbound in plasma, *K*(*B*/*P*) = blood to plasma ratio (previously determined), and *Cl*_*int,u*_ = unbound intrinsic clearance (µL/min/mg).

14 $$E_{H} = \frac{{Cl_{H} }}{{Q_{H} }}$$

*E*_*H*_ = Hepatic extraction ratio, *Cl*_*H*_ = hepatic clearance (L/min), and *Q*_*H*_ = hepatic blood flow (set to 1500 mL/min).

### In vivo evaluation

#### Snapshot-PK analysis

Concentration–time profiles and the corresponding in vivo pharmacokinetic parameters were obtained after a single oral dose of racemic TKK130, SAKK381, or SAKK394 was administered to female mice (Charles River Laboratories) infected with *Plasmodium* (*P.*) *berghei*. Each compound was investigated in two mice at doses of 3, 10, and 30 mg/kg TKK130, and 50 mg/kg SAKK381 and SAKK394. At 1, 4, and 24 h after dosing, blood spots were collected on filter paper. Following drying, the filter paper was frozen at −80 °C until further sample preparation. The dried blood spots were cut to a fixed diameter of 5.5 mm and extracted for 30 min at 2000 rpm with acetonitrile/water (80/20, *v*/*v*) containing an internal standard. The supernatant was evaporated to dryness under a gentle stream of nitrogen, and the residue was reconstituted in acetonitrile/water (40/60, *v*/*v*). LC–MS/MS was used to quantify unknown samples against a freshly prepared whole blood calibration curve on dried blood spots. The final calibration range was 1.56–800 ng/mL (TKK130) and 6.25–800 ng/mL (SAKK381 and SAKK394), including low, mid, and high concentration quality controls (*n* = 2 each). The maximum concentration (C_max_), time to maximum concentration (t_max_), and total exposure from 0 to 24 h (AUC_0−t_) were determined using PK-Sim^®^—Version 11.2 (Open Systems Pharmacology, GPLv2 license).

The animal experiments adhered to local and national regulations of laboratory animal welfare in Switzerland. The local authority (Veterinäramt Basel Stadt) approved the protocol (awarded permission no. 1731 and 2303).

#### Racemate separation

The extracted dried blood spot samples were evaporated to dryness at 37 °C under a gentle nitrogen stream. The residue was spiked with 200 µL of 0.5% triethylamine (Fisher Scientific GmbH, Loughborough, UK) in acetonitrile (*v*/*v*) and 20 µL of the chiral derivatization agent 2,3,4,6-tetra-*O*-acetyl-beta-glucopyranosyl isothiocyanate in acetonitrile (3 mg/mL) and shaken at room temperature for 30 min at 450 rpm [30]. The supernatant was evaporated at 37 °C under a nitrogen stream and reconstituted in 90/10 methanol/water (*v*/*v*). A corresponding freshly prepared calibration curve using dried blood spots was processed within a concentration range of 125–8000 ng/mL (SAKK381) and 125–16,000 ng/mL (TKK130 and SAKK394), including three quality control levels (500, 4000, and 6000/12,000 ng/mL (*n* = 2 each)). The total clearance was calculated separately for each enantiomer according to Eq. 15. Equation 16 defines the clearance ratio of enantiomer 2 relative to enantiomer 1.

15 $$Cl_{total} = \frac{f \times D}{{AUC_{0 - t} }}$$

*Cl*_*total*_ = Total clearance (mL/h), *f* = bioavailability, *D* = dose (ng), and *AUC*_*0-t*_ = total exposure from 0 to 24 h (ng h/mL).

16 $$Cl_{2/1} = { }\frac{{Cl_{total, en.2} }}{{Cl_{total, en.1} }}$$

$Cl_{2/1}$ = Clearance ratio of enantiomer 2 relative to enantiomer 1, $Cl_{total, en.2}$ = total clearance of enantiomer 2, and $Cl_{total, en.1}$ = total clearance of enantiomer 1.

### LC–MS/MS method for quantification

Sample quantification of the 3-HPAs was conducted using an LC–MS/MS system. Separation was performed using a Luna C18 (3 µm; 100 × 2.00 mm) HPLC column (applied for all in vitro/ex vivo assays) or an XSelect HSS T3 (2.5 µm; 4.6 × 150 mm) HPLC column (used for racemate quantification), under gradient conditions with 0.1% formic acid in MS-grade water as mobile phase A and 0.1% formic acid in MS-grade methanol as mobile phase B. The flow rate was set to 400 µL/min, and the column oven was maintained at 40 °C. The analytes were monitored by multiple reaction monitoring (MRM) in positive ionization mode. Further details are presented in Table 1.

**Table 1:** Structures and mass spectrometric transitions of 3-hydroxypropanamidines

3-HPA	Structure^a^	Monitored transition [m/z]
TKK130		463.0 → 309.97 852.2 → 169.0^b^
SAKK234		499.05 → 310.05
SAKK374		523.04 → 310.05
SAKK381		463.05 → 310.1 852.2 → 169.0^b^
SAKK394		539.12 → 213.1 928.2 → 213.2^b^
SAKK420		541.02 → 310.1

^a^as hydrochlorides

^b^transition used in racemate quantification

### PBPK model of TKK130

#### Murine PBPK model of TKK130

**Fig. 1:**
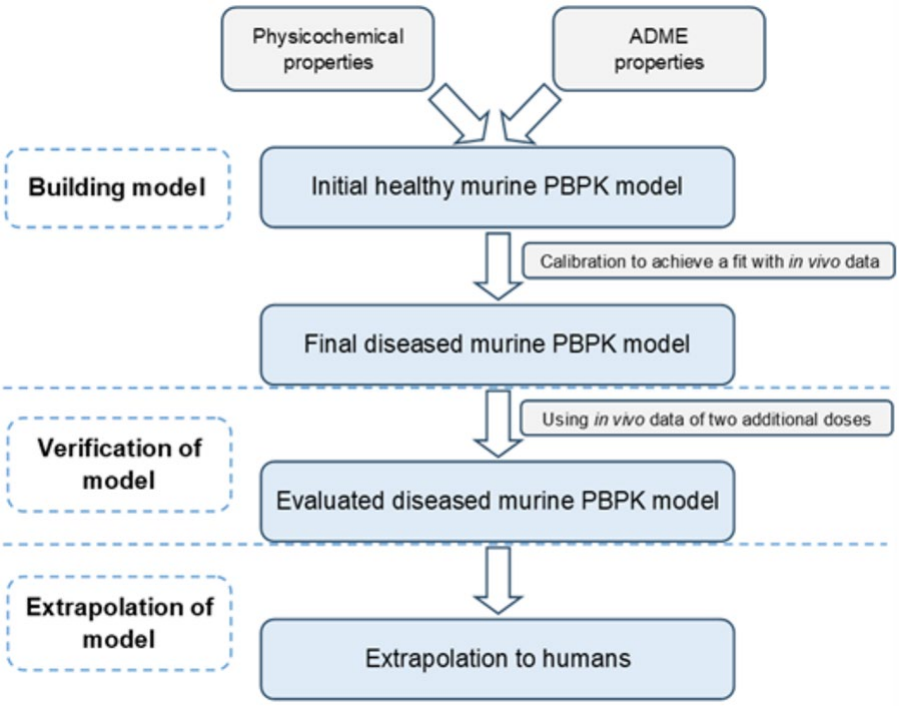
Workflow for building the PBPK model of TKK130

PBPK models were constructed in PK-Sim^®^ (Version 11.2) for the most promising 3-HPA TKK130. The initial model was developed for a single oral dose of 3 mg/kg in a murine population of 1000 animals, weighing between 15 and 25 g. The initial input parameters (Table 2) were either the results of the in vitro/ex vivo assays or, if unavailable, were calculated via ADMETlab 3.0 [31]. In addition, a sensitivity analysis was performed in PK-Sim^®^ using the 3 mg/kg mouse model to assess the influence of parameters derived from ADMETlab 3.0 and the pharmacokinetic assays on key pharmacokinetic parameters (C_max_, T_max_, and AUC_0−t_). For this sensitivity analysis, a variation range of 0.5, corresponding to a 50% change in the input parameters, was set [32]. The initial model in healthy mice was further extended to a diseased model using the corresponding in vivo data of TKK130 obtained from mice infected with *P. berghei* (see 2.3). Subsequently, the calibrated infectious disease model was verified using simulations of 10 and 30 mg/kg doses and their corresponding in vivo data (Fig. 1). The PBPK model is based on the “standard model for small molecules”, which incorporates four subcompartments per organ (compartments for blood cells, plasma, interstitial space, and cellular space). To ascertain the specific intestinal permeability, the determined P_app_ value was converted to the corresponding effective permeability coefficient (P_eff_) (Eq. 17).

**Table 2:** Input data and calculation methods of the initial TKK130 PBPK model

Value	Input data (initial)	Source/comment
*Physicochemical properties*		
Molecular weight	462.12 g/mol	
Effective molecular weight	360.12 g/mol	Molecular weight excluding covalently bound halogens^a^
Lipophilicity (log P)	5.23	Calculated by ADMETlab 3.0
Solubility	1.3 mg/L	Calculated by ADMETlab 3.0
p*K*a (base)	6.74	Calculated by ADMETlab 3.0
Fraction unbound in plasma	0.4%	Plasma protein binding assay (dilution method)
*ADME properties*		
Specific intestinal permeability	1.9 × 10^−6^ cm/s (P_app_)	Permeability assay (conversion into P_eff_ value necessary)
Total hepatic clearance	3.86 min^−1^	Microsomal stability assay
*Calculation methods*		
Partition coefficients		PK-Sim^®^ Standard
Cellular permeability		Charge dependent Schmitt

^a^required for PK-Sim^®^ to estimate permeability; considers the small contribution of halogens to the molecular volume in relation to their weight [33]

#### Human PBPK model of TKK130

For the first prediction of human pharmacokinetic behavior, the mouse model was extrapolated to a standard European population (weighing 53–93 kg, 50% female, 1000 individuals) using PK-Sim^®^ (Version 11.2). In accordance with the FDA, an equivalent human dose for initial clinical studies of the active and nontoxic dose in mice (30 mg/kg) was determined using Eq. 18 [18, 34]. The resulting equivalent dose was 2 mg/kg, based on a mean body weight of 73 kg. Simulations were conducted under fasted and fed states (1000 kcal meals) to evaluate the influence of food on TKK130 levels.

17 $$f = \frac{{P_{eff} \left( {literature} \right)}}{{P_{app} \left( {assay} \right)}}$$

*f* = Factor for conversion from P_app_ to P_eff_, *P*_*eff*_ (*literature*) = effective permeability coefficient from the literature (cm/s), and *P*_*app*_ (*assay*) = apparent permeability coefficient from the assay (cm/s).

18 $$HED = Dose(a) \times \left( {\frac{weight(a)}{{weight(h)}}} \right)^{0.33}$$

*HED* = Human equivalent dose (mg/kg), *Dose* (a) = animal dose (mg/kg), *weight* (a) = animal weight (kg), and *weight* (h) = human weight (kg).

## Results

### In vitro/ex vivo evaluation

#### Intestinal permeability

TKK130, the most hydrophilic 3-HPA, exhibited the highest permeability (P_app_ value: 1.9 ± 0.8 × 10^−6^ cm/s [mean ± SD]), followed by about a factor of 10 less for SAKK381 (P_app_ value: 1.2 ± 0.2 × 10^−7^). In contrast, none of the more lipophilic compounds (SAKK234, SAKK374, SAKK394, and SAKK420) were detectable on the acceptor side within 4 h under the applied assay conditions, and the P_app_ was assumed to be zero.

Notably, the 3-HPAs with larger substituents (i.e., bromine and phenyl) did not permeate in the selected setting. A correlation between the determined logD_7.4_ values and the P_app_ values was observed, yielding an *R*^2^ of 0.8698. In accordance with the Biopharmaceutical Classification System (BCS), all 3-HPAs (hydrochlorides) are classified as class 4 drugs with low solubility and low permeability. The detailed results of the calculated P_app_ values are presented in Table 3.

**Table 3:** Apparent permeability coefficients (P_app_) of the 3-hydroxypropanamidines, presented as the means ± standard deviations (*n* = 3)

Compound	P_app_ (cm/s)	Log D
TKK130	1.9 ± 0.8 × 10^−6^	4.4
SAKK234	–^a^	5.6
SAKK374	–^a^	5.4
SAKK381	1.2 ± 0.2 × 10^−7^	5.1
SAKK394	–^a^	5.6
SAKK420	–^a^	5.6

^a^No analyte was detected on the acceptor side in the assay

#### Plasma protein binding

**Fig. 2:**
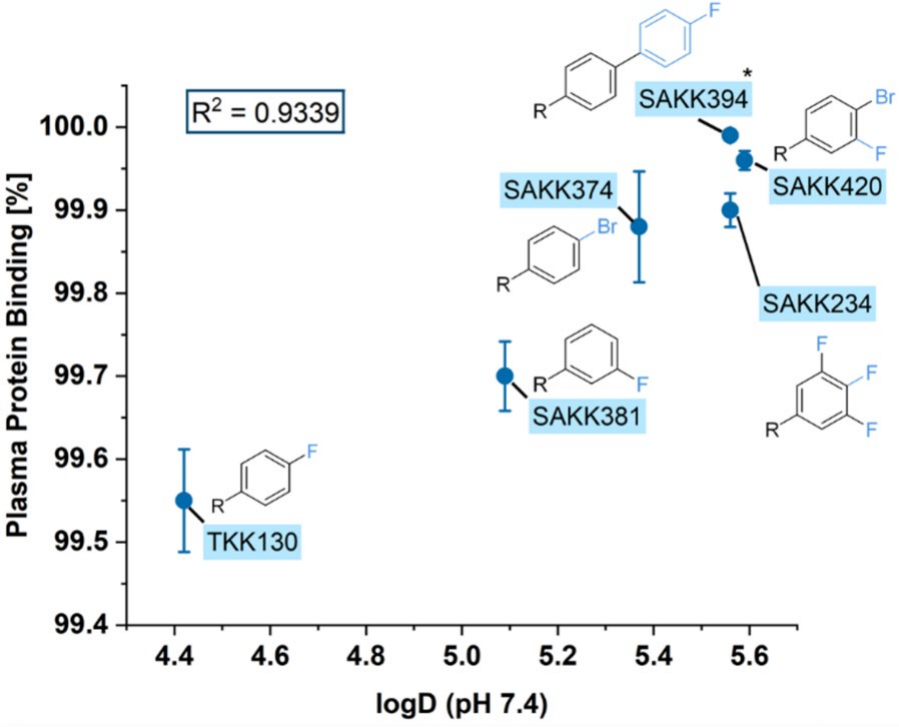
Correlation between the plasma protein binding of 3-hydroxypropanamidines and the log D at pH 7.4; values are presented as the mean ± standard deviation (*n* = 6). * *n* = 3 only

Applying equilibrium dialysis, plasma protein binding was found to be very high for all 3-HPA compounds (>99%). In line with EMA recommendations, the dilution method was additionally applied to accurately assess fractions unbound below 0.01 [27]. Among the investigated compounds, TKK130 (the most hydrophilic compound) exhibited the lowest plasma protein binding of 99.55 ± 0.06% (mean ± SD). Conversely, one of the most lipophilic compounds, SAKK394, demonstrated the highest plasma protein binding of 99.99 ± 0.00%. The present relationship is emphasized by a linear correlation between the logD and the plasma protein binding values with an *R*^2^ of 0.9339 (Fig. 2). Compounds with larger substituents exhibit higher plasma protein binding values. The detailed plasma protein binding values are compiled in Table 4.

**Table 4:** Plasma protein binding of 3-hydroxypropanamidines at two dilution levels, presented as the mean ± standard deviation

3-HPA	1:10 dilution (*n* = 3)	1:20 dilution (*n* = 3)	Mean
TKK130	99.56 ± 0.05	99.55 ± 0.08	99.55 ± 0.06
SAKK234	99.91 ± 0.02	99.89 ± 0.02	99.90 ± 0.02
SAKK374	99.91 ± 0.01	99.86 ± 0.02	99.88 ± 0.07
SAKK381	99.73 ± 0.02	99.67 ± 0.04	99.70 ± 0.04
SAKK394	Not available^a^	99.99 ± 0.00	99.99 ± 0.00^a^
SAKK420	99.97 ± 0.00	99.95 ± 0.00	99.96 ± 0.01

^a^Acceptor concentration below the lower limit of quantification

#### Blood-to-plasma ratio

**Fig. 3:**
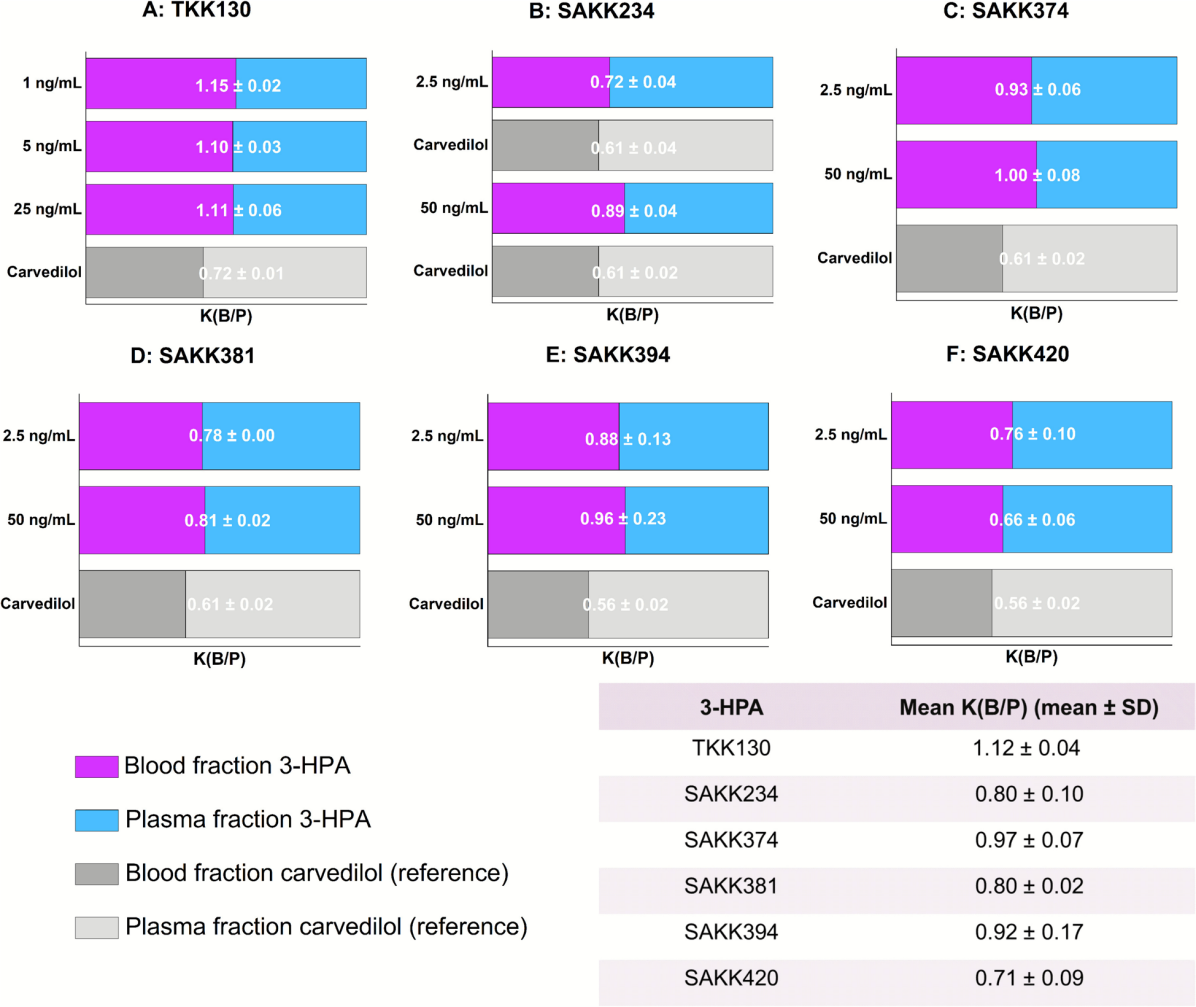
Blood-to-plasma ratios of 3-HPAs at different concentrations. **A** TKK130; **B** SAKK234; **C** SAKK374; **D** SAKK381; **E** SAKK394; **F** SAKK420. Carvedilol served as an assay reference. The data are presented as means ± standard deviations (*n* = 3 per concentration level). K(B/P) = blood-to-plasma ratio; 3-HPA = 3-hydroxypropanamidine; SD = standard deviation

To evaluate the distribution of 3-HPAs between the blood and the plasma, the blood-to-plasma ratios were determined. A value of less than 1 indicates a preference for distribution in the plasma, whereas a K(B/P) of greater than 1 indicates a preference for distribution in the red blood cells. TKK130, with a mean value of 1.12 ± 0.04, displays a slight preference for distribution within red blood cells, whereas SAKK381 (0.80 ± 0.02), SAKK234 (0.80 ± 0.10), and SAKK420 (0.71 ± 0.09) exhibit a preference for distribution in plasma. The values for SAKK374 (0.97 ± 0.07) and SAKK394 (0.92 ± 0.17) indicate a distribution between plasma and red blood cells that is approximately equal, with K(B/P) values of about 1. All 3-HPAs exhibited a concentration-independent distribution. Further details are presented in Fig. 3.

#### Microsomal stability and hepatic extraction ratio

**Fig. 4:**
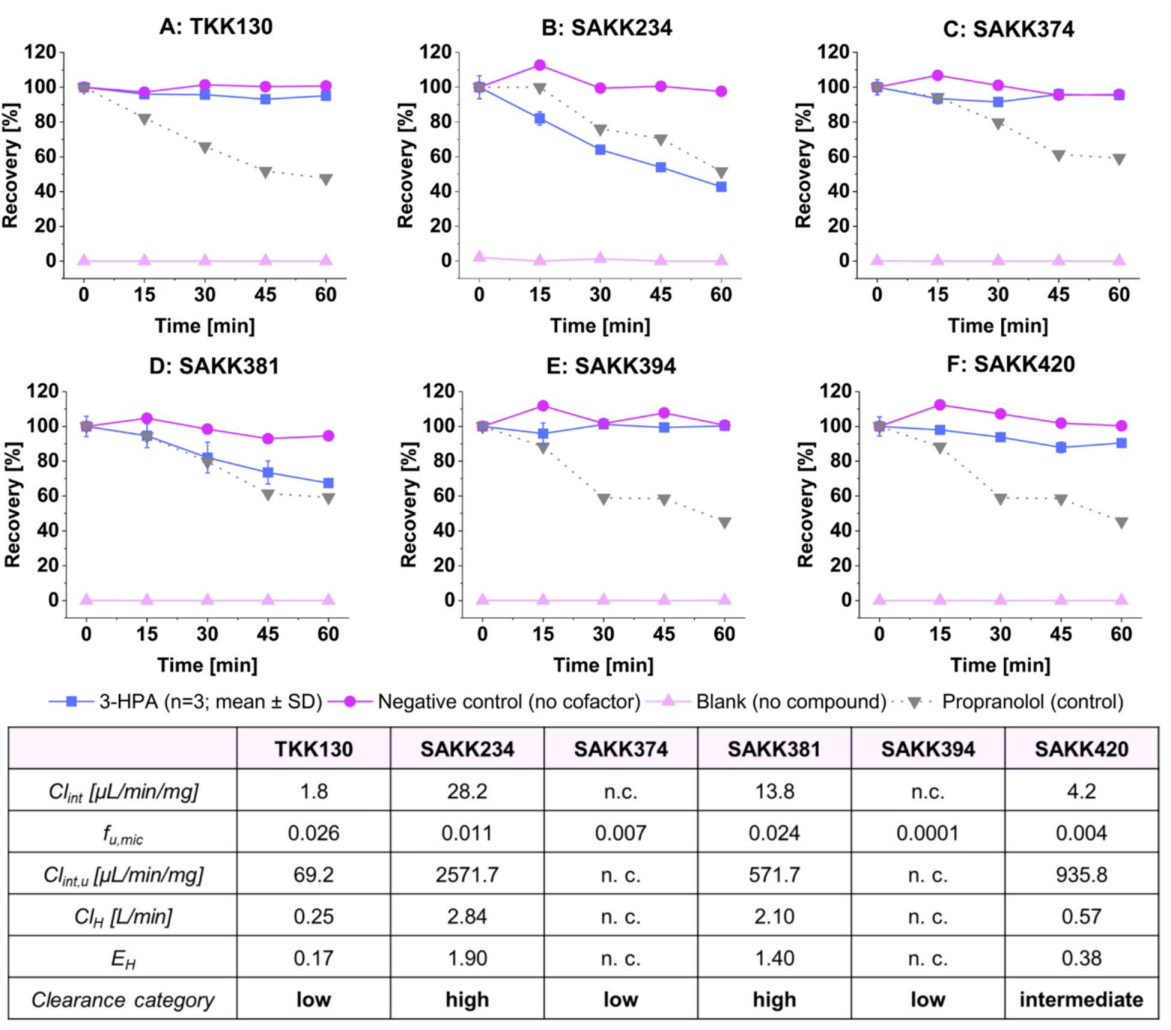
Microsomal stability of TKK130 (**A**), SAKK234 (**B**), SAKK374 (**C**), SAKK381 (**D**), SAKK394 (**E**), and SAKK420 (**F**) observed in human liver microsomes (pooled from 150 donors); mean ± SD (*n* = 3); including a negative control (no cofactor) and a blank (no compound); propranolol served as assay reference; intrinsic clearance (Cl_int_), fraction unbound in microsomes (f_u,mic_), unbound intrinsic clearance (Cl_int,u_), hepatic clearance (Cl_H_), hepatic extraction ratio (E_H_) and clearance category of 3-HPAs. n.c. = not calculable; 3-HPA = 3-hydroxypropanamidine; SD = standard deviation

3-HPAs were metabolized to varying degrees by HLMs. While SAKK374 and SAKK394 were not degraded, SAKK234 presented the highest intrinsic clearance (Cl_int_) of 28.2 µL/min/mg among all the compounds. The values for Cl_int_ were corrected for nonspecific binding to human liver microsomes [27], enabling the calculation of the hepatic extraction ratio and the classification of the compounds into low- (TKK130, SAKK374, and SAKK394), intermediate- (SAKK420), and high- (SAKK234 and SAK381) extraction drugs. SAKK374 and SAKK394 were identified as low-extraction drugs, as no degradation by HLMs was observed, but free fractions were present. Figure 4 shows the detailed degradation of the compounds in the HLMs and the calculation of the (unbound) intrinsic clearance, hepatic clearance, and hepatic extraction ratio.

#### Summary of the in vitro/ex vivo PK results

An initial evaluation based on pharmacokinetic parameters of six different 3-HPAs was conducted using the results of the in vitro/ex vivo assays. The two most hydrophilic 3-HPAs, TKK130, a low-extraction drug, and SAKK381, a high-extraction drug, demonstrated promising pharmacokinetic properties (i.e., due to the highest intestinal permeability and lowest PPB). Notably, TKK130 displayed a K(B/P) ratio exceeding 1, indicating a slight preference for the distribution into red blood cells, which is considered relevant for 3-HPAs, given their targeting of the heme detoxification pathway [18].

Conversely, the low-extraction drug SAKK394 showed high lipophilicity, limited permeability, and the highest PPB, suggesting an unfavorable in vivo profile.

These three representative compounds were selected for further in vivo analyses to confirm the meaningfulness of the in vitro/ex vivo datasets.

### In vivo evaluation

#### Snapshot-PK analysis

**Fig. 5:**
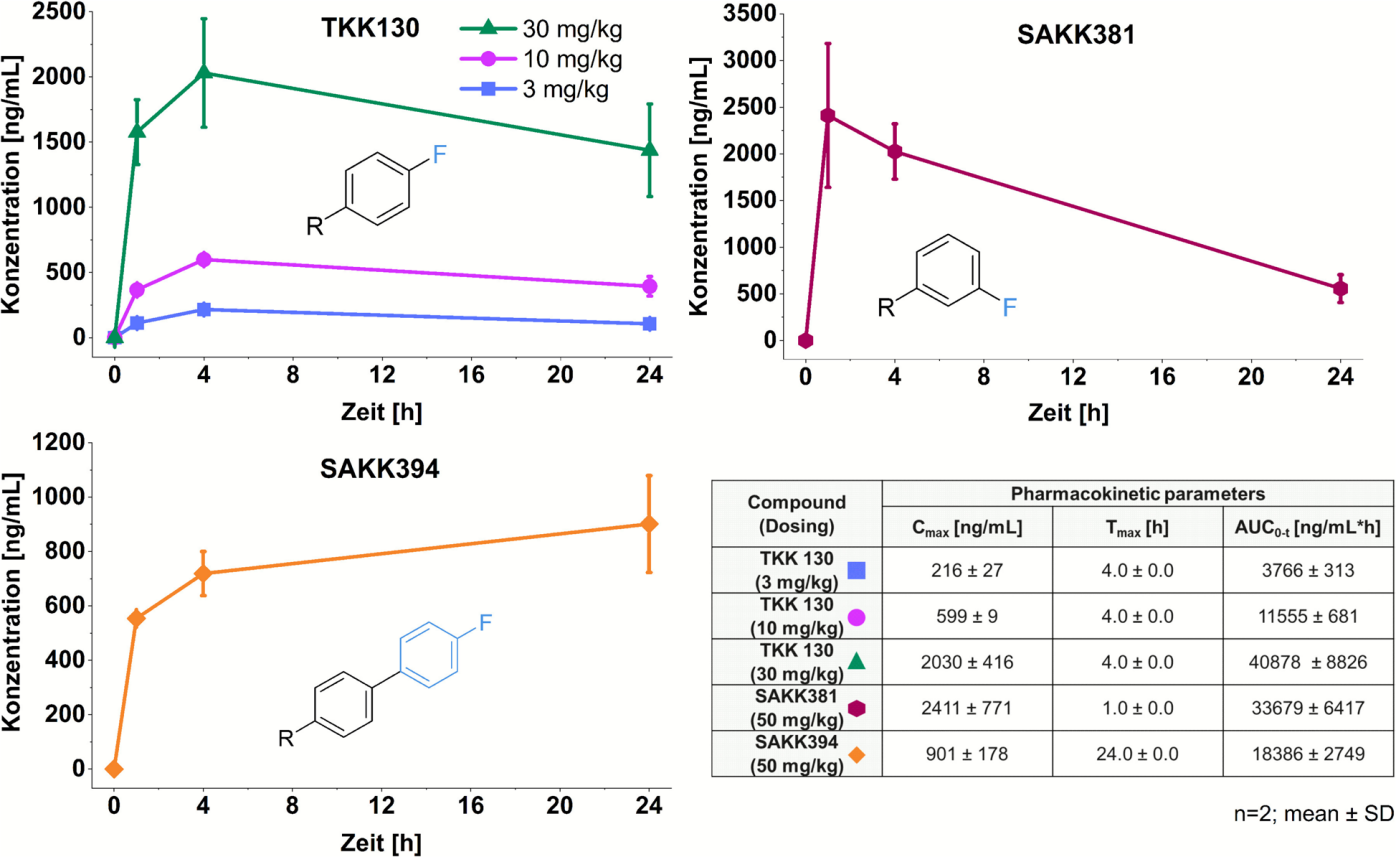
In vivo snapshot pharmacokinetics of TKK130, SAKK381, and SAKK394 in *Plasmodium berghei*-infected mice treated with single oral doses of 3, 10, and 30 mg/kg (TKK130) and 50 mg/kg (SAKK381 and SAKK394); concentrations over time are presented as the means ± SDs (*n* = 2). C_max_ = maximum concentration; T_max_ = time to maximum concentration; AUC_0−t_ = total exposure from 0 to 24 h; SD = standard deviation

TKK130 demonstrated a long-lasting profile in *Plasmodium berghei* mice and an almost linear relationship between the three different doses. At all doses, the maximum concentrations were attained at 4 h postadministration. The corresponding AUC_0−t_ ranged between 3766 ng/mL h (3 mg/kg) and 40,878 ng/mL h (30 mg/kg). SAKK381 showed faster uptake with the highest concentration of 2411 ng/mL (mean) at 1 h postadministration (dose: 50 mg/kg), an AUC_0−t_ of 33,679 ng/mL h, and a profound decrease. Conversely, SAKK394 exhibited a markedly prolonged increase in blood concentration, with a C_max_ of 901 ng/mL (mean) determined at 24 h and an AUC_0−t_ of 18,386 ng/mL h. The detailed concentration–time profiles and the C_max_, t_max_, and AUC_0−t_ values are compiled in Fig. 5.

The results of this snapshot-PK analysis are in line with the findings of the in vitro/ex vivo evaluation. The most hydrophilic TKK130 exhibited the highest dose-adjusted AUC_0−t_, which is consistent with its highest permeability and low liver metabolism. The PK profile of SAKK381 is associated with relatively high intestinal permeability and high metabolism of liver enzymes. SAKK394, the compound with the lowest solubility, no measurable intestinal permeability, and low liver metabolism, presented a pharmacokinetic profile with a very slow increase in blood concentration over time.

#### Racemate separation

**Fig. 6:**
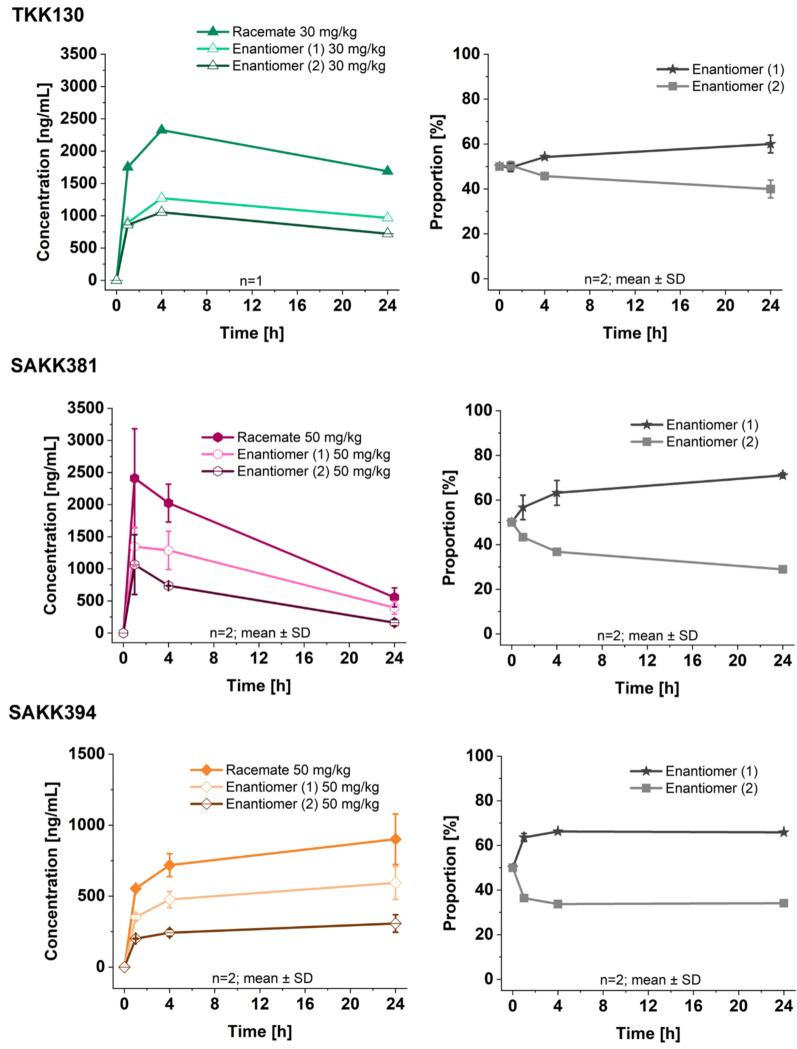
Racemate separation of TKK130, SAKK381, and SAKK394; left: concentrations over time in *Plasmodium berghei*-infected mice for the racemate and both enantiomers for doses of 30 mg/kg (TKK130, *n* = 1) and 50 mg/kg (SAKK381 + SAKK394, *n* = 2); right: proportion of both enantiomers over time (*n* = 2, for TKK130 mean of 10 and 30 mg/kg (each *n* = 1), data for 10 mg/kg not shown). SD = standard deviation

In the absence of enantiomerically pure standards, peak 1 was designated as enantiomer 1 and peak 2 was designated as enantiomer 2. Figure 6 shows the PK profiles displaying the concentrations of the racemate and the calculated concentrations of both enantiomers (left) and the proportions of both enantiomers over time (right).

The analysis of the snapshot PK samples of racemic TKK130, SAKK381, and SAKK394 revealed an alteration toward a greater availability of enantiomer 1 over time. After 24 h, the proportion of enantiomer 1 was 60.0 ± 4.0% (mean ± SD) for TKK130, 71.0 ± 1.1% for SAKK381, and 65.9 ± 0.1% for SAKK394. The calculated total clearance values were greater for enantiomer 2 than for enantiomer 1. For TKK130, the clearance was 41.2 ± 5.0 mL/h for enantiomer 2, whereas it was 32.2 ± 2.5 mL/h for enantiomer 1, corresponding to a clearance ratio of 1.2. For SAKK381, the clearances were 97.6 ± 7.9 mL/h for enantiomer 2 and 56.7 ± 11.5 mL/h for enantiomer 1, resulting in a clearance ratio of 1.7. In the case of SAKK394, the clearance of enantiomer 2 was 175.5 ± 27.4 mL/h, whereas that of enantiomer 1 was 90.9 ± 15.5 mL/h, resulting in a clearance ratio of 1.9.

#### Overall evaluation

In conclusion, TKK130 was identified as the compound with the most favorable pharmacokinetic profile, i.e., exhibiting the highest hydrophilicity, intestinal permeability, plasma unbound fraction, and distribution into red blood cells, as well as a low level of hepatic metabolism among the compounds studied. In vivo, TKK130 has been shown to achieve the highest C_max_ and AUC_0−t_ values, as well as the lowest enantioselective clearance among all investigated 3-HPAs, along with the highest antiplasmodial activity and no detectable toxicity in *Plasmodium berghei*-infected mice [18, 35]. Klein et al. reported that TKK130 achieved a 100% cure rate (3/3 mice) at a dose of 4 × 30 mg/kg, whereas SAKK381 and SAKK394 had no curative effect even at higher doses (4 × 50 mg/kg; 0/3 mice each). Based on those published pharmacodynamic data and the pharmacokinetic results presented here, TKK130 was identified as the most promising 3-HPA derivative and was subsequently subjected to further in silico evaluation.

### PBPK-model of TKK130

#### Murine PBPK model of TKK130

Using the initial input data (Table 2), the model did not demonstrate an adequate fit to the in vivo data regarding the absorption and elimination processes. Therefore, the model was calibrated using the in vivo data from the snapshot-PK analysis. First, the absorption phase was adjusted by the conversion of the P_app_ value to the P_eff_ value based on literature values for the reference compounds. The final conversion value of 5.5 × 10^−6^ cm/s for the specific intestinal permeability was within the designated range. Second, additional clearance processes were necessary to accurately model the elimination phase observed in vivo. The addition of a biliary clearance achieved appropriate degradation. In the absence of a biliary clearance, the PBPK model demonstrates a markedly slower elimination profile than that observed in mice. This results in a delay in the t_max_ (i.e., 4.8 vs. 7.8 h at 3 mg/kg), as well as an increase in the C_max_ (i.e., 232 vs. 320 ng/mL at 3 mg/kg) and AUC_0−t_ (i.e., 4095 vs. 6846 ng/mL h at 3 mg/kg). The final set of input parameters, following calibration, and the calculation methods employed are presented in Table 5. The sensitivity analysis revealed that the LogP, pKa, and the specific intestinal permeability had the most influence on C_max_, T_max_, and AUC_0−t_ (Supplementary Table 1).

**Table 5:** Final input parameters and calculation methods of the TKK130 PBPK model

Value	Input parameters (final)
*Physicochemical properties*	
Molecular weight	462.12 g/mol
Effective molecular weight	360.12 g/mol
Lipophilicity (log P)	5.23
Solubility	1.3 mg/L
p*K*a (base)	6.74
Fraction unbound in plasma	0.4%
*ADME properties*	
Specific intestinal permeability	5.5 × 10^−6^ cm/s (P_eff_)
Total hepatic clearance	3.86 min^−1^
Biliary clearance	110 min^−1^
*Calculation methods*	
Partition coefficients	PK-Sim^®^ Standard
Cellular permeability	Charge dependent Schmitt

**Fig. 7:**
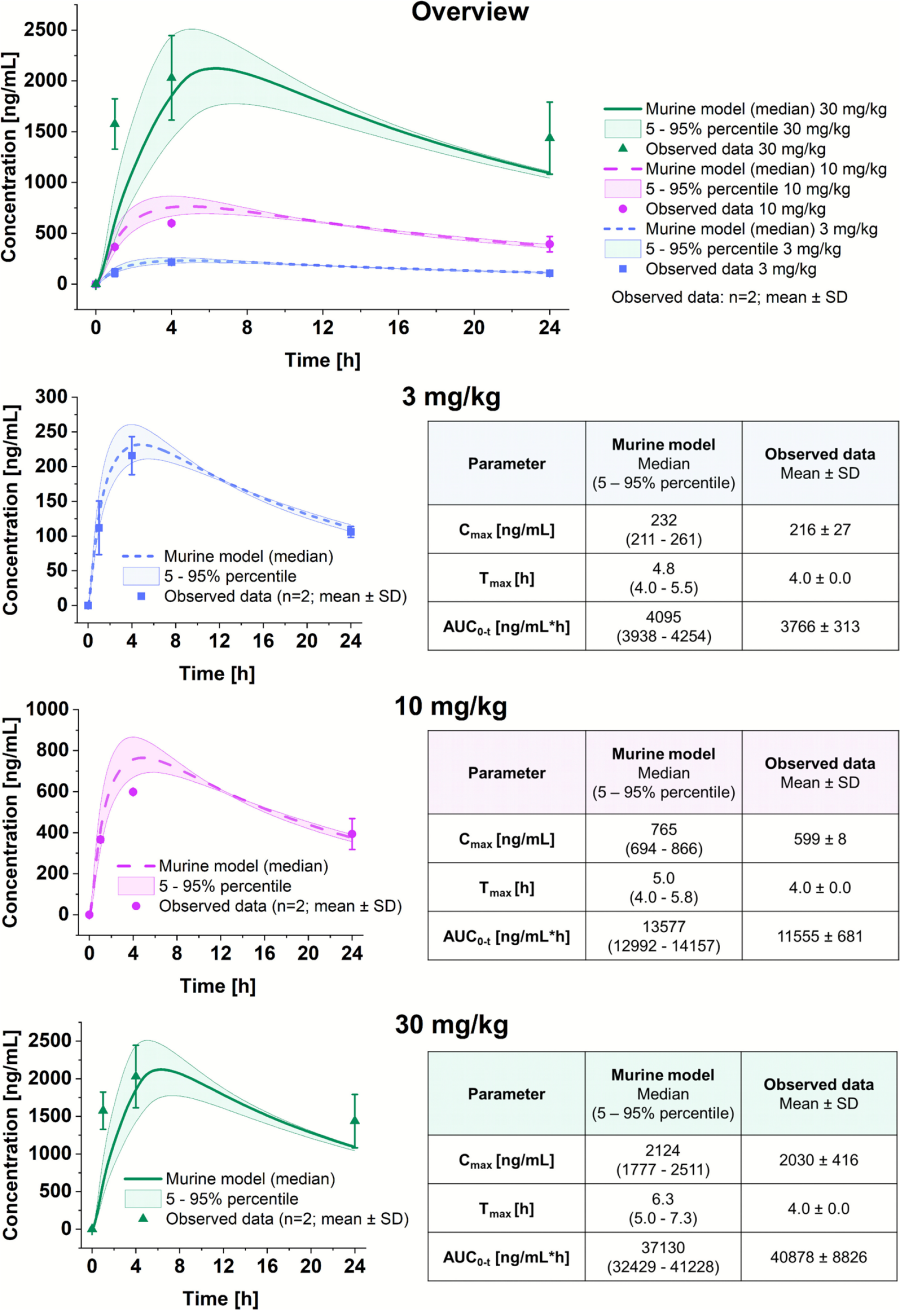
Murine diseased PBPK models of TKK130 in populations of 1000 individuals in whole blood for doses of 3, 10, and 30 mg/kg shown as medians with 5–95% percentiles; observed data were obtained in *Plasmodium berghei*-infected mice (*n* = 2) and are shown as the means ± SDs. C_max_ = maximum concentration; T_max_ = time to maximum concentration; AUC_0−t_ = total exposure from 0 to 24 h; SD = standard deviation

**Fig. 8:**
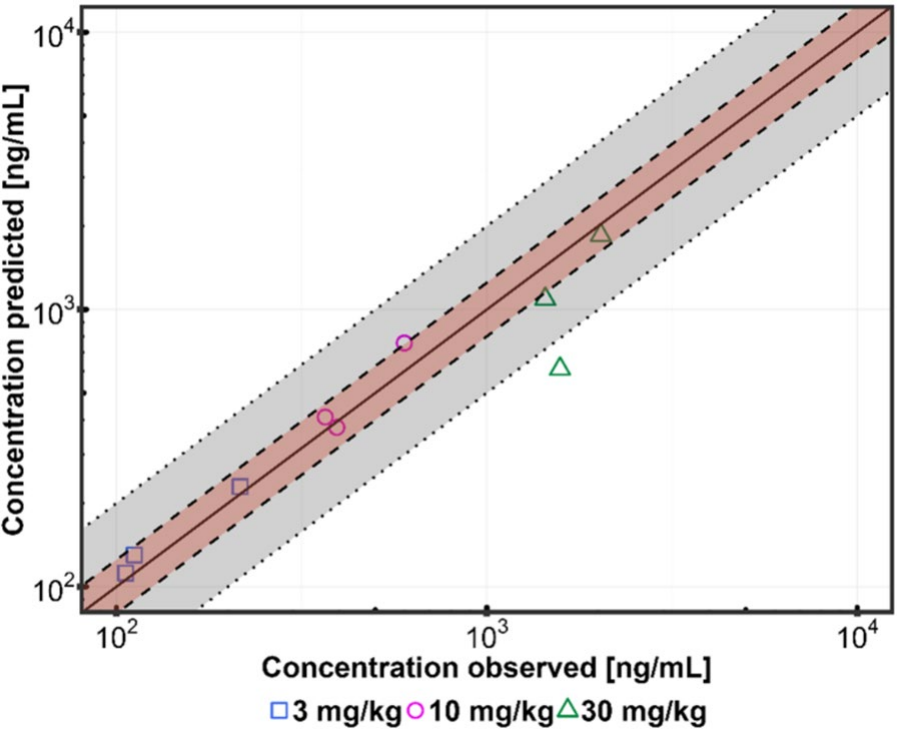
Goodness-of-fit plot for the TKK130 PBPK model; shown are predicted versus observed concentrations for the three doses 3 mg/kg, 10 mg/kg, and 30 mg/kg; the red area shows the 0.8–1.25-fold interval (dashed line); the gray area shows the 0.5–2.0-fold interval (dotted line)

Following calibration of the initial healthy murine model, the final diseased model of TKK130 was verified using the observed in vivo data. The model showed a good fit of the predicted data to the observed data for all three doses (3 mg/kg, 10 mg/kg, and 30 mg/kg), as demonstrated by the C_max_, t_max_, and AUC_0−t_ values being within a twofold range of the in vivo data (Fig. 7). The AUC_0−t_ and C_max_ of the in vivo PK data are within the 5–95% percentile of the predicted 3 mg/kg and 30 mg/kg doses, but below the 5–95% percentile of the 10 mg/kg dose. The measured t_max_ in mice at 3 mg/kg and 10 mg/kg remains within the lower range of the 5–95% percentile (4.0 h vs. 4.0–5.0 h and 4.0–5.8 h, respectively) but is below the range for the 30 mg/kg model (4.0 h vs. 5.0–7.3 h). The goodness-of-fit plot (Fig. 8) demonstrates that the 3 mg/kg data were within the 0.8–1.25-fold interval of the observed blood concentrations, and data for 10 mg/kg are within the 0.5–2-fold interval. The same applies to most of the data points at 30 mg/kg (except at 1 h postdose). Overall, the evaluated and verified diseased model is of high quality, with the corresponding in vivo data almost completely falling within the specified interval.

#### Human PBPK model of TKK130

To make an initial prediction of pharmacokinetics in humans, the murine model was extrapolated to a human population (weighing 53–93 kg, 50% female, 1000 virtual individuals). The model predicted a C_max_ of 60 ng/mL (median; 5–95% percentile: 18.2–189.5 ng/mL), a t_max_ of 4.5 h (median; 5–95% percentile 2.5–8.3 h), an AUC_0−t_ of 1846 ng/mL h (median; 5–95% percentile: 814–3856 ng/mL h), and a half-life of 80.5 h (median; 5–95% percentile: 53.7–132.8 h) in the fasted state. The predictions indicated high variability in TKK130 between individuals. A comparison of the fasted and fed states revealed a substantial increase in the fraction absorbed (50% fasted vs. 99% fed). Likewise, the predicted fed state values for C_max_ (median 131; 5–95% percentile 43–255 ng/mL) and AUC_0−t_ (median 3407; 5–95% percentile: 1633–5264 ng/mL h) also increased approximately twofold. Additionally, the predicted values for t_max_ (median 6.8 h; 5–95% percentile 3.8–11.3 h) and the half-life (98.0 h; median; 5–95% percentile: 61.4–182.1 h) were slightly higher in the fed state. Additionally, interindividual variation differed only slightly between the fasted and fed states.

**Fig. 9:**
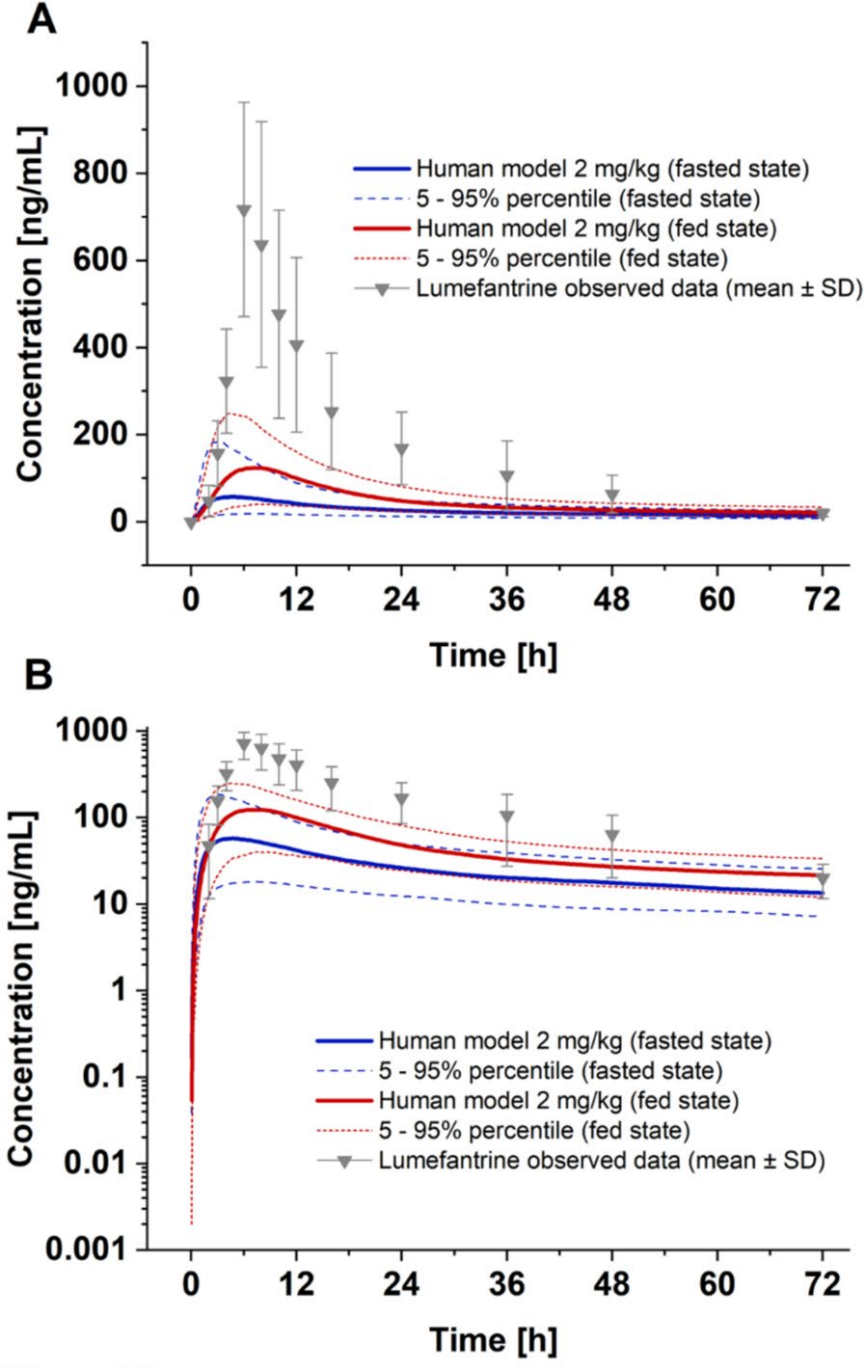
First, human pharmacokinetic prediction based on the extrapolation of the verified murine model for a dose of 2 mg/kg TKK130 in whole blood, shown as the median with the 5–95% percentile under fasted and fed states; for comparison, dose-adjusted data from a pharmacokinetic clinical study of lumefantrine in humans (fed state) are presented [36]. **A **Linear *y*-axis, **B** logarithmic *y*-axis

The predicted time-concentration profile in whole blood under fasted and fed states, as well as data from a pharmacokinetic clinical study of lumefantrine in healthy humans under the fed state (as a reference), are shown in Fig. 9.

## Discussion

Applying a preclinically guided sorting approach of the novel class of 3-HPAs against malaria provided (1) initial pharmacokinetics, (2) facilitated the ranking for further in vivo testing on the basis of in vitro/ex vivo Drug Metabolism and Pharmacokinetics (DMPK) data, and (3) allowed for preliminary prediction in humans using the developed PBPK model. TKK130 was identified as the most promising compound because of its favorable in vitro, ex vivo, and in vivo pharmacokinetics. Considering its efficacy, high cure rates, and lack of signs of toxicity in mice, TKK130 is an excellent candidate for further preclinical and clinical development against malaria.

Malaria is an infectious disease caused by protozoan parasites of the genus *Plasmodium*. *Plasmodium falciparum*, the most prevalent species in Africa and Southeast Asia, is responsible for most fatalities worldwide. The WHO recommends artemisinin-based combination therapies (ACTs) for treating uncomplicated *P. falciparum* malaria, with artemether/lumefantrine constituting 75% of the African market [37–39]. The combination of artemether and lumefantrine enhances the antiparasitic effect, as artemether kills the parasites and lumefantrine prevents them from spreading further. However, resistance to ACTs has increased, as reflected by failure rates of up to 19.5% for artemether/lumefantrine in the WHO African region [37, 40]. New drug candidates are highly needed for the combination treatment of malaria due to increasing levels of resistance. A promising class of 3-HPAs was developed as alternative long-acting partner drugs to fast-acting artemisinin derivatives.

Compared with the well-established long-acting lumefantrine, CYP interactions may play a minor role in the administration of the low-extraction drugs TKK130, SAKK374, and SAKK394, and the intermediate-extraction drug SAKK420. The determined Cl_int_ values in HLMs were comparable to those of lumefantrine (Cl_int_ < 7 µL/min/mg), whereas the high-extraction drugs SAKK381 and SAKK234 were more comparable to halofantrine (Cl_int_ 26.9 µL/min/mg) [41]. Furthermore, notable differences were observed between the individual 3-HPAs investigated, which are closely correlated with their logD_7.4_ values and their chemical structure, particularly the sizes of the different substituents: PPB was the lowest, and intestinal permeability was the highest for the more hydrophilic TKK130 and SAKK381 with the smallest substituents. Overall, the in vitro/ex vivo DMPK data of TKK130 and SAKK381 appear highly promising from a pharmacokinetic perspective, with SAKK381 exhibiting greater clearance. Notably, TKK130 had a K(B/P) ratio exceeding 1, indicating a slight preference for distribution into red blood cells, which is considered relevant for 3-HPAs, given their targeting of the heme detoxification pathway [18].

The AUC is regarded as the most important parameter of lumefantrine in relation to the 28-day outcome and has been identified as exhibiting a positive correlation with the cure rate [36]. Preliminary in vivo pharmacokinetics in *P. berghei* mice revealed the highest dose-adjusted total exposure over time for TKK130 (AUC_0−t_ TKK130: 1500 ± 89 µmol min/L (mean ± SD); AUC_0−t_ lumefantrine: 1381 ± 302 µmol min/L), followed by SAKK381 (875 ± 167 µmol min/L) and SAKK394 (410 ± 61 µmol min/L) [42]. These findings were anticipated by ex vivo data, which revealed the highest intestinal permeability, the lowest plasma protein binding, and minimal metabolism by HLMs for TKK130. In mice, TKK130 already demonstrated a complete cure rate (100%) after a maximum investigation period of 30 days (3 out of 3 mice; 4 × 30 mg/kg), comparable to that observed with lumefantrine [18]. Survival after the administration of SAKK381 (15 days, 4 × 50 mg/kg) and SAKK394 (10 days, 4 × 50 mg/kg) was substantially shorter, and no mouse was cured [35].

Moreover, lumefantrine undergoes rapid elimination, with plasma concentrations declining to near zero within 24 h [36]. This has resulted in an extensive dosing regime of lumefantrine (0, 8, 24, 36, 48, and 60 h) [36, 43, 44]. While SAKK381 had a similar elimination rate, TKK130 exhibited slower degradation, potentially allowing for a less complex dosing regimen and maybe improved patient adherence.

The results collectively identified TKK130 as the most promising compound because of its favorable in vitro/ex vivo and in vivo profiles. Consequently, a PBPK model for TKK130 was developed to gain initial insights into its pharmacokinetic behavior in humans.

The murine model demonstrated good predictive performance for all tested doses concerning AUC_0−t_, C_max_, and t_max_. At the highest dose, the model predicted delayed absorption compared with the 3 and 10 mg/kg dose models and the observed in vivo data. This deviation may be attributed to the limited number of animals and sampling time points in the in vivo study, prolonged absorption at higher doses due to limited solubility, or the use of the nonionic solution enhancer Tween^®^ 80 in the drug formulations [18]. Since the effect of the enhancer cannot be represented by the model, this may explain the discrepancy in the observed t_max_. Nevertheless, all the predicted values fell within the 0.5–2-fold range of the observed data, except for the 1 h concentration at 30 mg/kg, which is consistent with the later predicted t_max_ compared with the observed data. The data to build the model was obtained from a drug screening for snapshot-PK of multiple promising novel drug candidates. Accordingly, this snapshot-PK study was designed to provide a first insight into the in vivo PK behavior rather than a statistically powered evaluation. Higher numbers of animals could strengthen the test power of the model, reduce uncertainty in the pharmacokinetic data, and therefore provide a more accurate basis for creating the model. Although the obtained data showed low interindividual variability, future preclinical studies will require larger animal cohorts to refine the PBPK model prior to clinical trials in humans.

The sensitivity analysis revealed that logP, pKa, and specific intestinal permeability had the greatest impact on the simulated pharmacokinetic parameters and consequently on the model output. The fact that logP and pka did not require adjustment during the calibration process supports the robustness of the estimations obtained using ADMETlab. Furthermore, these results suggest that the literature-based conversion method applied to the permeability assay data yields valid and reliable results.

The model’s extrapolation to humans revealed a pharmacokinetic profile characterized by high interindividual variability at the calculated effective dose. This variability can be attributed to the high lipophilicity of TKK130 and its differential distribution based on the body’s fat content. Nevertheless, the predicted whole blood concentrations in humans are all below the concentrations observed in mice, where no signs of toxicity were seen [18]. The dose-adjusted blood concentrations of the structurally similar drug lumefantrine in humans exceeded the predicted levels for TKK130. However, as there are no real human pharmacokinetic data available at the moment, the model can only provide a hint of how pharmacokinetic behavior in humans would be. For validation, real clinical data must be generated to prove the accuracy of the human model. Nevertheless, the model can serve as a starting point for first-in-human dose predictions and provides an initial overview of pharmacokinetics in humans. The high interindividual variability aligns with the findings reported for lumefantrine, which also demonstrated high variability in its pharmacokinetic profile in humans [45]. Additionally, a food effect was predicted, resulting in a more than twofold increase in total exposure of TKK130. This observation is in line with lumefantrine [43, 44] and is beneficial for combination therapy with the fast-acting artemether, which is recommended to be administered with a high-fat meal. Data from future preclinical and clinical studies of TKK130 will be integrated into the model to increase its validity for adults and to extend its applicability to children, who are most affected by malaria. The PBPK model provides a framework for the development of future PBPK models of promising 3-HPAs, in which compound-specific parameters such as solubility, permeability, and clearance can be integrated.

The modeling approach has several advantages over the single-species allometric scaling approach. PBPK models incorporate several physiological data, i.e., enzyme configuration, tissue composition, and species-specific characteristics. Especially in preclinical development and before first-in-human dose trials, PBPK models can be particularly useful for gaining more knowledge about a drug. This could help minimize the failure of drugs in early clinical development and optimize clinical and preclinical trials [46, 47]. Data from future (pre)clinical studies will be integrated into the model to enhance its validity for adults and to extend its applicability to children, who are most affected by malaria.

Overall, the applied selection strategy based on physiologically relevant DMPK assays adapted for high lipophilicity, followed by selected in vivo studies in mice, identified TKK130 as the most promising compound due to its favorable in vitro, ex vivo, and in vivo pharmacokinetics. Considering its efficacy, high cure rates, and no signs of toxicity in mice, TKK130 is an excellent candidate for further preclinical and clinical development.

## Conclusions

The presented preclinically guided selection approach was successful in identifying TKK130 as the most promising compound of novel antimalarial 3-hydroxypropanamidines because of its favorable in vitro/ex vivo and in vivo pharmacokinetics. The construction of a PBPK model enabled the initial prediction of TKK130 pharmacokinetics in humans, indicating high interindividual variability and a relevant impact of food. Combined with its excellent in vivo efficacy and lack of observed toxicity in mice, TKK130 emerges as a highly promising candidate for future preclinical and clinical investigations.

## Supplementary Information

Additional file 1.

## Data Availability

The datasets used and/or analyzed during the current study are available from the corresponding author on reasonable request.

## Abbreviations

3-HPA(s): 3-Hydroxypropanamidine(s)
ACTs: Artemisinin-based combination therapies
ADME: Absorption, distribution, excretion, metabolism
AUC_0__−__t_: Area under the curve/total exposure from 0−t h
BCS: Biopharmaceutical classification system
BSA: Bovine serum albumin
Cl_H_: Hepatic clearance
Cl_int_: Intrinsic clearance
C_max_: Maximum concentration
DMPK: Drug metabolism and pharmacokinetics
DMSO: Dimethyl sulfoxide
E_H_: Hepatic extraction ratio
EMA: European Medicines Agency
ESI: Electrospray ionization
FaSSIF-V2: Fasted state simulating intestinal fluid—version 2
FDA: U.S. Food and Drug Administration
F_u,mic_: Fraction unbound in microsomes
LC–MS/MS: High-performance liquid chromatography coupled with tandem mass spectrometry
HLMs: Human liver microsomes
HPLC: High-performance liquid chromatography
K(B/P): Blood-to-plasma ratio
MRM: Multiple reaction monitoring
MS: Mass spectrometry
*m*/*z*: Mass-to-charge ratio
*P**.*: *Plasmodium*
P_app_: Apparent permeability coefficient
PBPK: Physiologically based pharmacokinetic
PBS: Phosphate-buffered saline
PK: Pharmacokinetic(s)
P_eff_: Effective permeability coefficient
RBC: Red blood cells
SD: Standard deviation
T_max_: Time to maximum concentration
