# Supplementary material for: Integrative preclinical strategy for pharmacokinetic profiling and candidate selection of 3-hydroxypropanamidines: a promising antimalarial class
Source: Malar J. 2026 Mar 10;25:151. doi: 10.1186/s12936-026-05837-9 (PMC13064256; doi:10.1186/s12936-026-05837-9)
Supplement: Supplementary file 1 — Additional file 1. [file 12936_2026_5837_MOESM1_ESM.docx]

Supplementary Table 1: Sensitivity analysis for the 3 mg/kg TKK130 mouse PBPK model with a variation range of 0.5

| Parameter | PK-Parameter | Value |
| --- | --- | --- |
| Lipophilicity (logP) | C_max_ | -5.0 |
|  | T_max_ | 7.4 |
|  | AUC_0-t_ | 0.1 |
| Solubility | C_max_ | 0.0 |
|  | T_max_ | 0.0 |
|  | AUC_0-t_ | 0.0 |
| pKa (base) | C_max_ | -0.8 |
|  | T_max_ | 5.0 |
|  | AUC_0-t_ | -0.7 |
| Fraction unbound in plasma | C_max_ | -0.1 |
|  | T_max_ | 0.0 |
|  | AUC_0-t_ | -0.1 |
| Specific intestinal permeability | C_max_ | 0.5 |
|  | T_max_ | -0.9 |
|  | AUC_0-t_ | 0.2 |
| Total hepatic clearance | C_max_ | 0.0 |
|  | T_max_ | 0.0 |
|  | AUC_0-t_ | 0.0 |
| Biliary clearance | C_max_ | -0.2 |
|  | T_max_ | -0.2 |
|  | AUC_0-t_ | -0.4 |
